# The DNA-Based Authentication of Commercial Herbal Products Reveals Their Globally Widespread Adulteration

**DOI:** 10.3389/fphar.2019.01227

**Published:** 2019-10-24

**Authors:** Mihael Cristin Ichim

**Affiliations:** “Stejarul” Research Centre for Biological Sciences, National Institute of Research and Development for Biological Sciences, Piatra Neamt, Romania

**Keywords:** herbal products, food supplements, traditional medicines, authentication, adulteration, contamination, DNA

## Abstract

The herbal products, sold worldwide as medicines or foods, are perceived as low risk because they are considered natural and thus safe. The quality of these products is ineffectively regulated and controlled. The growing evidence for their lack of authenticity is causing deep concern, but the scale of this phenomenon at the global, continental or national scale remains unknown. We analyzed data reporting the authenticity, as detected with DNA-based methods, of 5,957 commercial herbal products sold in 37 countries, distributed in all six inhabited continents. Our global survey shows that a substantial proportion (27%) of the herbal products commercialized in the global marketplace is adulterated when their content was tested against their labeled, claimed ingredient species. The adulterated herbal products are distributed across all continents and regions. The proportion of adulterated products varies significantly among continents, being highest in Australia (79%), South America (67%), lower in Europe (47%), North America (33%), Africa (27%) and the lowest in Asia (23%). The commercial HPs’ authenticity among the 37 countries included in our global analysis ranges between 0 and 100% from the total number of product reported for each specific national marketplace. For 9 countries, more than 100 products were successfully DNA-based authenticated and reported. From these countries, the highest percentage of adulterated commercial HPs was reported for Brazil (68%), followed distantly by Taiwan (32%), India (31%), USA (29%), followed closely by Malaysia (24%), Japan (23%), South Korea (23%), Thailand (20%), and China (19%). Our results confirm the large-scale presence of adulterated herbal products throughout the global market. The adulterated herbal products contain undeclared contaminant, substitute, and filler species, or none of the labeled species, which all may be accidental or intentional, economically-motivated and fraudulent. Due to the ever-increasing analytical sensitivity of the high throughput DNA sequencing, increasingly used for the untargeted, simultaneous multi-taxa identification, the proportion of adulterated HPs detected on the global market is expected to increase. In the context of the increasing demand for HPs, the limited supply of raw materials derived from many plant species, some of which being already nationally or internationally protected and having various degrees of trade restrictions, adds up to the differences and discrepancies between national HPs’ regulatory frameworks and further increases the risks of adulteration of many types of herbal products. The globally widespread adulteration is a serious threat to consumers’ well-being and safety, in spite of herbal products’ claimed or expected health benefits.

## Introduction

Based on traditional medicinal knowledge, developed and refined over centuries of empirical testing, the herbal products (HPs) are perceived as low risk because they are considered natural and thus safe (Jordan et al., 2010; Raclariu et al., 2018). The HPs are known under many terms, such as herbal drugs, botanical drugs, botanicals, phytomedicines, traditional medicines (TMs), herbal medicines (HMs), traditional Chinese medicines (TCMs), traditional herbal medicinal products (THMPs), natural health products (NHPs) or plant food supplements (PFSs). Their names depend on their final declared use (i.e., medicines or foods) and the prevailing national legal frameworks and regulatory requirements (Simmler et al., 2018). Even more diverse are the forms under which the HPs are commercialized: free-dried herbs, teas, extracts, decoctions, infusions, poultices, essential oils, tinctures, glycerites, powders, pills, tablets, capsules, drops, softgels, syrups, etc. The HPs are used in herbal medicine (HM), also called botanical medicine, herbalism, phytomedicine, or phytotherapy, and it refers to herbs, herbal materials, herbal preparations, and finished herbal products that contain parts of plants or other materials as active ingredients used for medicinal purposes. Herbal medicine (HM) is a core part of traditional medicine (TM), complementary and alternative medicine (CAM) or traditional and complementary medicine (T&CM), these terms being used interchangeably, depending on the country. World Health Organization (WHO) has recognized TM as a truly global phenomenon, which is only growing and expanding, particularly with respect to products bought in person (over-the-counter) or over the Internet (e-commerce) (World Health Organization, 2013; Mezzasalma et al., 2017). WHO estimates that more than 100 countries worldwide have regulations for herbal medicines, although in many cases, they significantly differ, so counterfeit, poor quality, or adulterated herbal products in international markets are serious patient safety threats (Wheatley and Spink, 2013; Osathanunkul et al., 2018).

Authentic HPs are by definition non-adulterated (Simmler et al., 2018). Accidental or deliberate adulteration (Simmler et al., 2018) includes contamination, use of fillers and product substitution (Shanmughanandhan et al., 2016). The traditional pharmacopoeial identification methods for authentication include botanical taxonomy, macroscopic and microscopic examination, and chemical methods (Pawar et al., 2017). Macroscopic and microscopic identity examinations may fail when a product consists of botanicals that have been processed beyond the ability to provide morphological characterization. Chemical analysis of specific marker compounds encounters problems when these compounds are not distinct to a given species or when purified reference standards are not available (Pawar et al., 2017). With the rapid advances of DNA sequencing technologies, the detection of species-specific DNA sequences, *i.e.* DNA barcoding, is already officially recognized as identification method (Pharmacopoeia Committee of P. R. China, 2015; British Pharmacopoeia Commission, 2018). Nevertheless, in spite of all available regulations and authentication methods, mislabeled, adulterated and counterfeit HPs are persistent in commercial markets (Gao et al., 2017), some of them having chronic, acute, or even lethal, adverse health effects (Posadzki et al., 2013).

The HPs’ lack of authenticity is documented but the extent of this phenomenon at a global, continental or national scale remains unknown in spite of an increasing body of scientific evidence. An accurate estimation is of paramount importance for buyers, consumers or patients, scientists, as well as for the individuals and entities along the HPs’ value chains (Booker et al., 2012), such as regulators, growers, collectors, processors, producers, traders, distributors, exporters, importers, retailers, pharmacists, traditional healers and medical practitioners.

## Results

**Figure 1:**
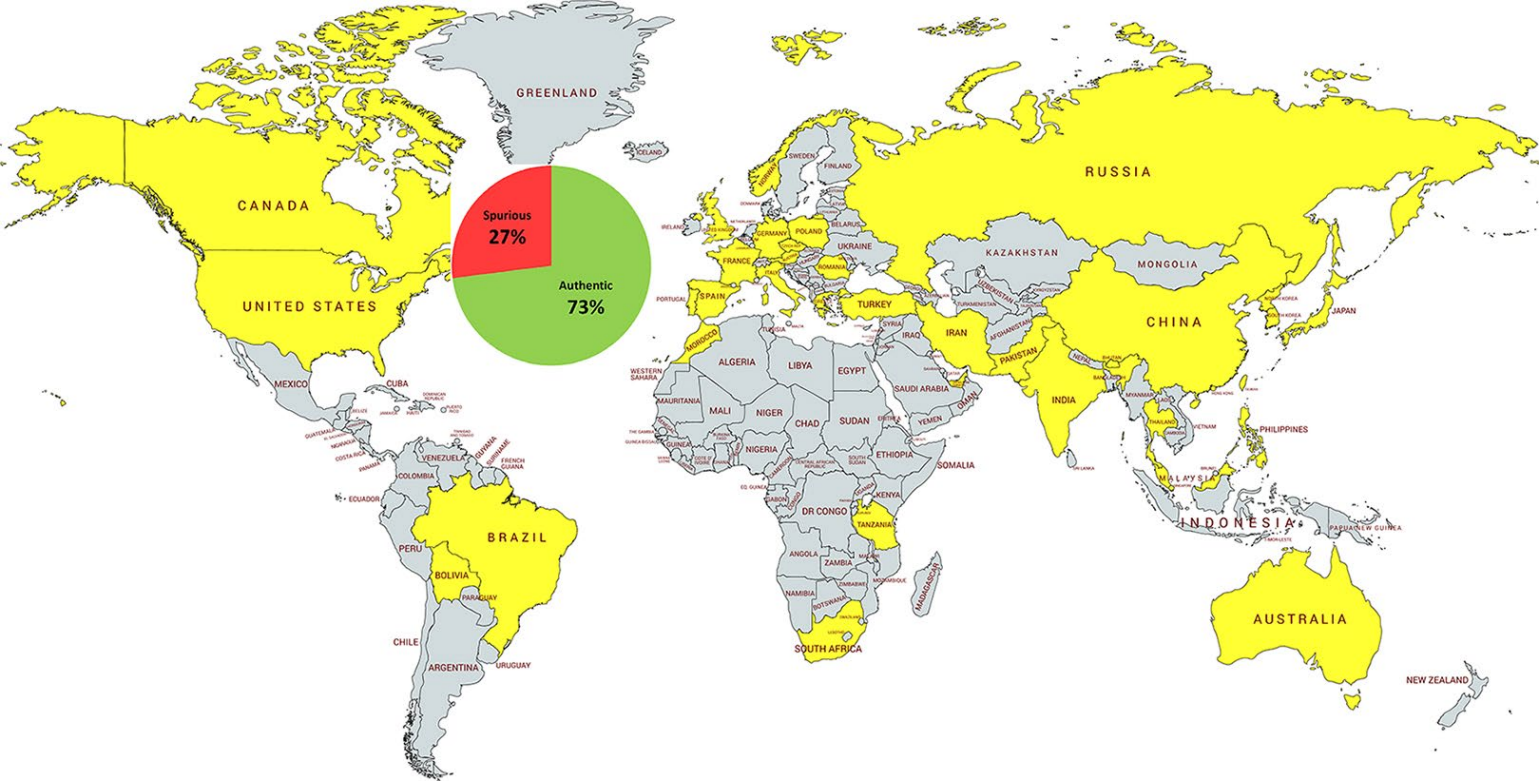
The distribution of the DNA-tested herbal products and their overall authenticity at global level. The HPs were sold in 37 countries (yellow) on six continents: Asia (16), Europe (13), Africa (3), North America (2), South America (2), and Australia (1). Countries not included in the analysis are shaded gray.

Here we present the first global survey of the HPs’ authenticity. We analyzed the results reported for 5,957 commercially available herbal products sold in 37 countries, distributed across all six inhabited continents (except Antarctica) (**Figure 1**).

Their authenticity was tested with various species-specific DNA-based methods, ranging from classical marker tools, to DNA barcoding, and DNA metabarcoding. Overall, 27% of all herbal products from the global marketplace were found to be adulterated (**Table 1**).

**Table 1:** The authenticity of commercial herbal products at continental and global level.

No.	Continent	Countries (no.)	Products (no.)	Products/country (X̃)	Authentic products		Adulterated products	
					No.	%^(*)^	No.	%^(*)^
1	Asia	16	4,807	300.4	3,694	77	1,113	23
2	Europe	13	293	22.6	154	53	139	47
3	Africa	3	119	59.5	87	73	32	27
4	North America	2	520	260	347	67	173	33
5	South America	2	155	77.5	51	33	104	67
6	Australia	1	63	63	13	21	50	79
	Total	37	5,957	161.2	4,356	73	1,611	27

*The percentage values were rounded to the nearest whole number.

**Figure 2:**
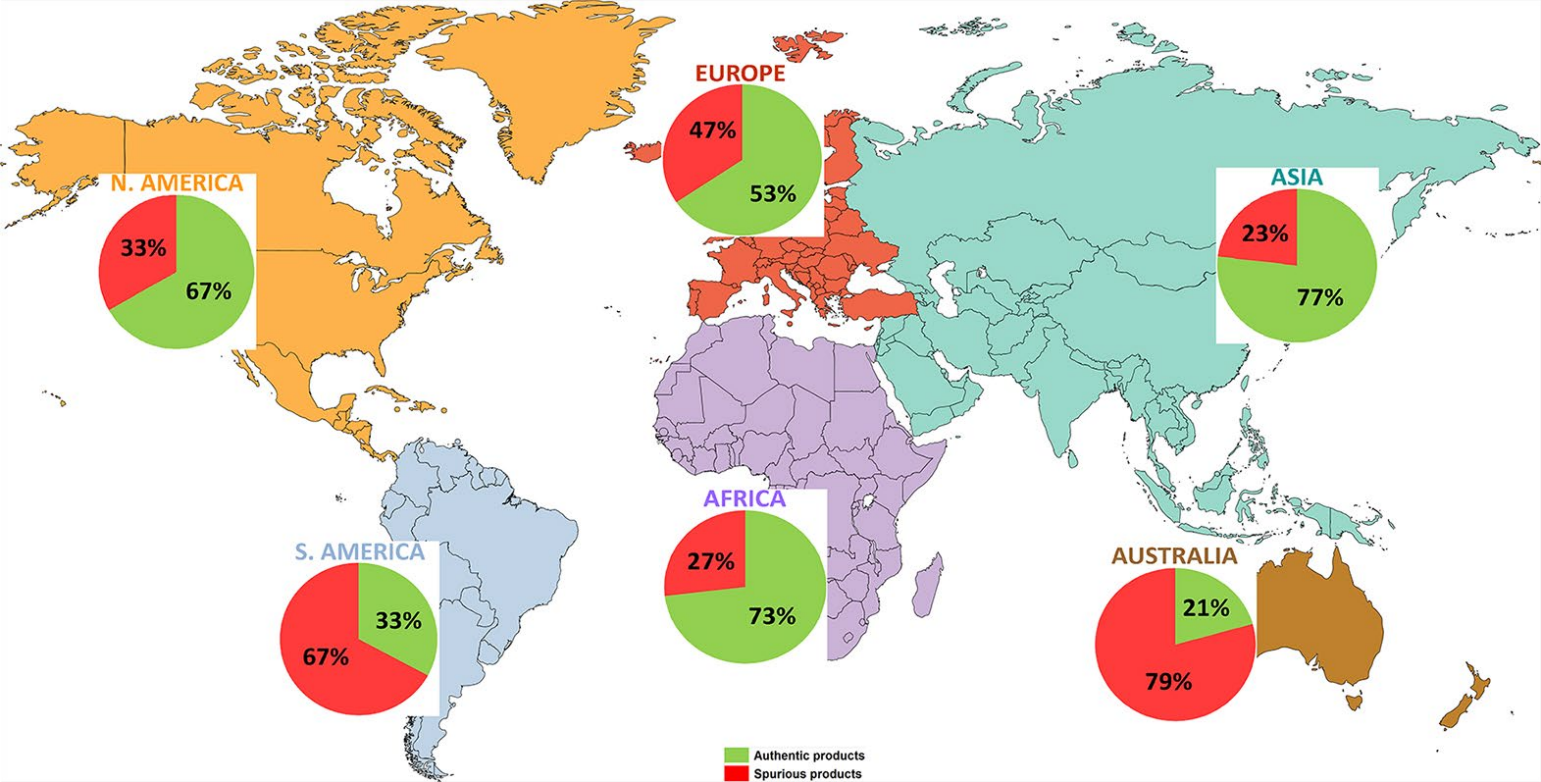
The distribution of the DNA-tested herbal products and their overall authenticity at continental level. The HPs’s DNA-based authenticity varies substantially among continents (authentic/adulterated %): Asia (77/23%) (light blue-green), Europe (53/47%) (reddish-orange), Africa (73/27%) (violet), North America (67/33%) (orange), South America (33/67%) (blue), and Australia (21/79%) (brown).

The adulterated herbal products were containing undeclared substitute, contaminant, filler species, or none of the labeled species at all. The proportion of adulterated products varies significantly among continents, being highest in Australia (79%), South America (67%), lower in Europe (47%), North America (33%), Africa (27%) and the lowest in Asia (23%) (**Figure 2** and **Table 1**). Only the percentage of adulterated herbal products sold in Asia is slightly lower than the global value (27%) while the percentage reported for Africa equals the global one. On these two continents, the traditional herbal medicines are widely used and sometimes are the only treatments available (World Health Organization, 2013). Asia, the continent with the lowest number of products reported as adulterated (23%), has instead the highest proportion (4/5) of products being analyzed with DNA-based methods from the total, the other five continents accounting together for the remaining one fifth of the products (**Table 1**).

At national level, the number of samples reported for each country varies even wider (**Table 2** and **Supplementary Table S1**). China is by far the best represented country with 2,809 commercial samples successfully authenticated, almost half (47%) of the samples DNA-tested from the entire global marketplace while India follows distantly with 752 commercial herbal products DNA-based authenticated. This suggests that the importance of these products in Asia, widely used in their traditional medicine, such as the traditional Chinese medicine (TCM) and the Ayurvedic medicine, is reflected by the interest of the scientific community in developing DNA-based methods for the authentication of these medicinal products. Directly influenced by some rapidly-spreading and highly influential traditional medicine systems in the Western countries, follows USA for which the authenticity results were reported for 465 herbal products.

**Table 2:** The authenticity of commercial herbal products sold on national and global market.

No.	Country	Products (no.)	Authentic products		Overall authenticity		Adulterated products	
					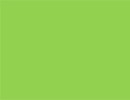	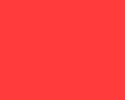		
			No.	%^(*)^	Authentic	**Adulterated**	No.	%^(*)^
1.	Bhutan	2	2	100	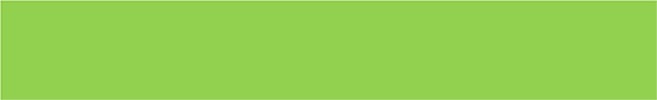		0	0
2.	China	2,809	2,271	81	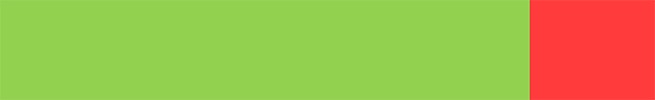		538	19
3.	Hong Kong	1	1	100	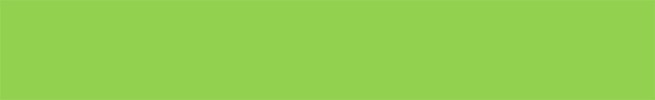		0	0
4.	India	752	517	69	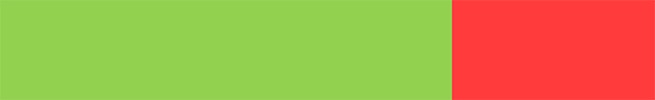		235	31
5.	Iran	72	52	72	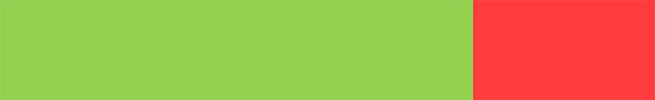		20	28
6.	Japan	162	125	77	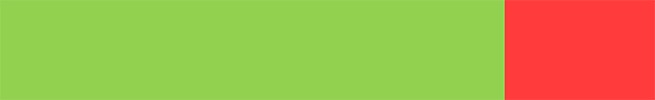		37	23
7.	Malaysia	136	104	76	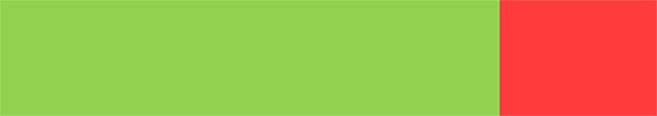		32	24
8.	North Korea	2	2	100	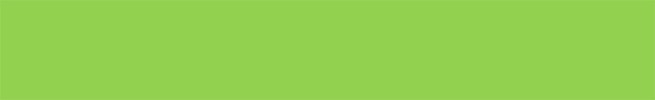		0	0
9.	Pakistan	36	29	81	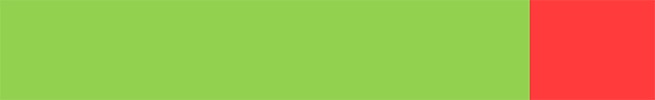		7	19
10.	Philippines	27	9	33	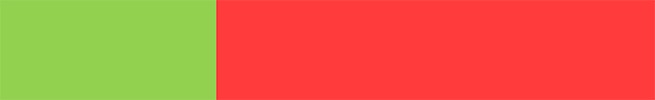		18	67
11.	Russia	6	0	0	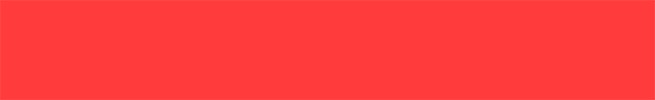		6	100
12.	Singapore	1	0	0	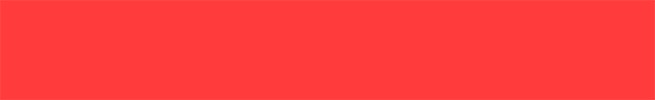		1	100
13.	South Korea	212	163	77	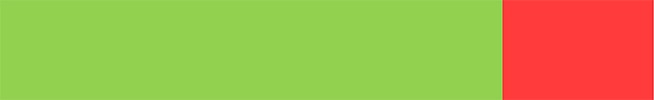		49	23
14.	Taiwan	453	309	68	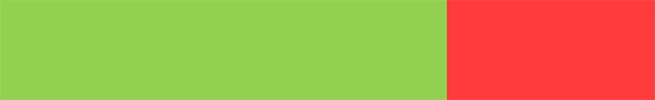		144	32
15.	Thailand	118	94	80	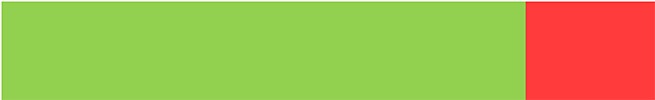		24	20
16.	United Arab Emirates	18	16	89	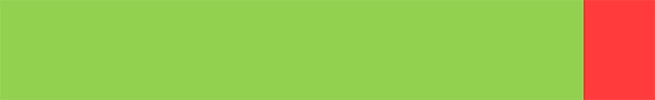		2	11
17.	Austria	13	10	77	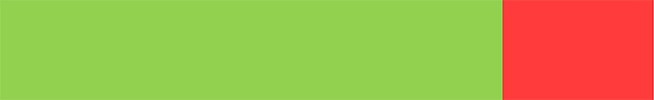		3	23
18.	Czech Republic	3	0	0	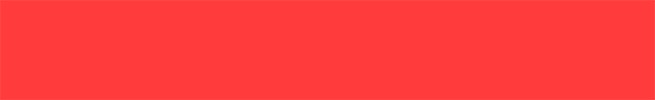		3	100
19.	France	1	0	0	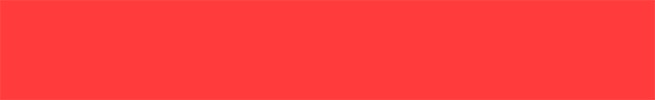		1	100
20.	Germany	29	13	45	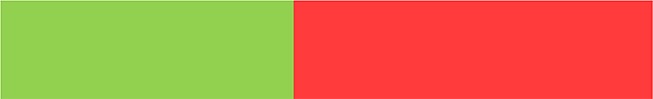		16	55
21.	Greece	8	7	87	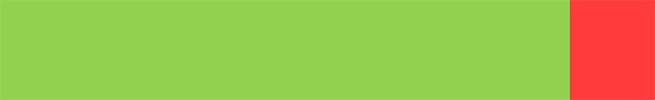		1	13
22.	Italy	55	44	80	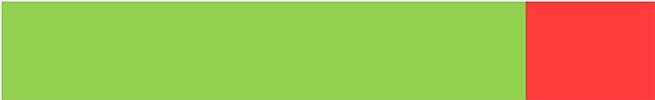		11	20
23.	Norway	3	0	0	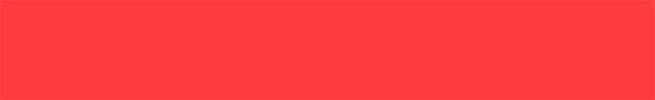		3	100
24.	Poland	5	1	20	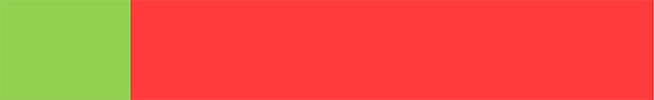		4	80
25.	Portugal	12	10	83	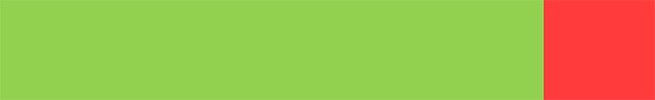		2	17
26.	Romania	70	4	6	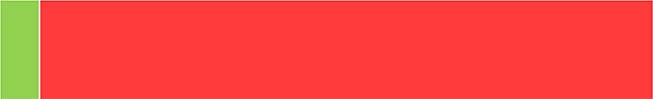		66	94
27.	Spain	2	1	50	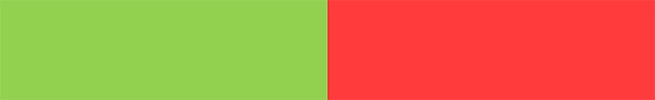		1	50
28.	Turkey	33	26	79	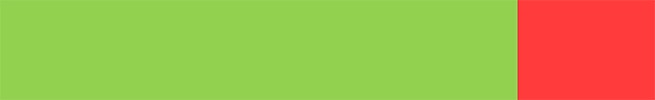		7	21
29.	United Kingdom	59	38	64	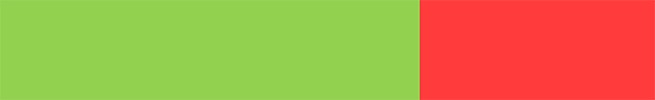		21	36
30.	Morocco	83	68	82	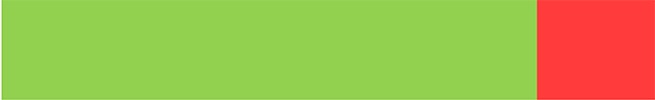		15	18
31.	South Africa	30	19	63	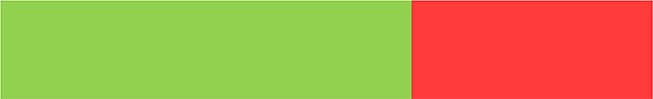		11	37
32.	Tanzania	6	0	0	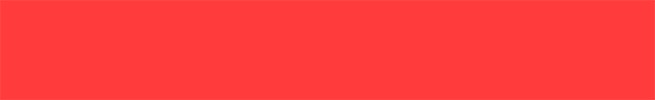		6	100
33.	Canada	55	16	29	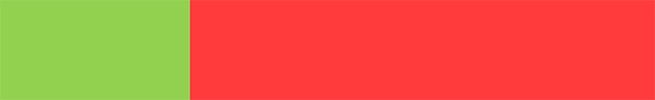		39	71
34.	USA	465	331	71	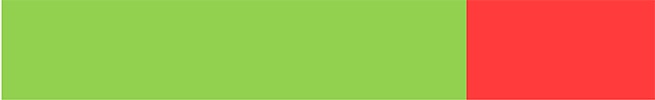		134	29
35.	Bolivia	1	1	100	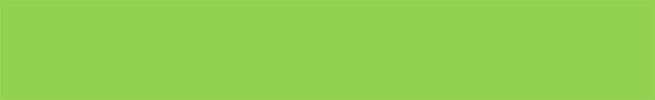		0	0
36.	Brazil	154	50	32	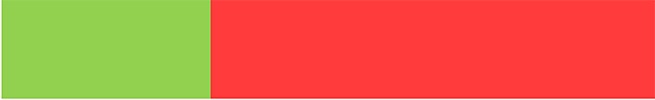		104	68
37.	Australia	63	13	21	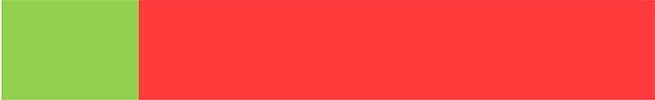		50	79
	TOTAL	5,957	4,346	73	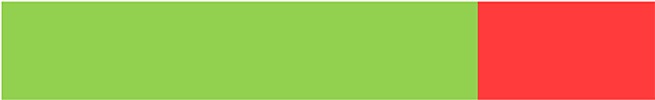		1,611	27

*The percentage values were rounded to the nearest whole number.

The HPs’ authenticity among the 37 countries included in our analysis ranges between 0 and 100% from the total number of product reported for each specific national marketplace. For 9 countries, distributed on three continents, more than 100 products were successfully DNA-based authenticated, and they account together for 88% of all samples tested and reported worldwide. From these countries, the highest percentage of adulterated commercial HPs was reported for Brazil (68%), followed distantly by Taiwan (32%), India (31%), USA (29%), followed closely by Malaysia (24%), Japan (23%), South Korea (23%), Thailand (20%), and China (19%).

Out of the total of 37 countries included in our global analysis, 10 of them (*e.g.*, Russia, Tanzania, Czech Republic, Norway, Bhutan, North Korea) have all their HPs tested either authentic or adulterated but with no more than 6 samples reported for each of them, while 4 countries have only one commercial HP reported after DNA-tested for authenticity (*i.e.*, Hong Kong, Singapore, France, and Bolivia).

## Discussion

Our global survey shows that 27% of all successfully analyzed commercial herbal products are not authentic when their content was tested with DNA-based analytical methods against their labeled, claimed, and expected composition. The adulterated HPs are distributed across all continents and regions. Accidental contamination through plant misidentification or cross-contamination during processing as well as the intentional and fraudulent use of cheaper substitute and filler species adds up to product misrepresentation, poor packaging or inappropriate labeling (Jordan et al., 2010; Osathanunkul et al., 2018). Although some monographs for herbal raw materials, such as those in the European Pharmacopoeia (Ph. Eur.) and the United States Pharmacopeia (USP), allow a certain amount (e.g., 2% in USP) of foreign organic matter (Parveen et al., 2016), it represents an accidental contamination while the presence of substitute or filler species are intentional, economically motivated and fraudulent.

A correlation, to some extent, exists between the percentage of adulterated HPs and the type of DNA-based method employed to analyze them. The classical DNA marker-based methods are targeted approaches aiming to detect the presence of certain species, usually the labeled ones (Mezzasalma et al., 2017). DNA barcoding, which makes use of short, standardized regions of the genome as species “barcodes” (Hebert et al., 2003), is a DNA Sanger sequencing-based targeted approach appropriate for testing single ingredient HPs (Newmaster et al., 2013; Raclariu et al., 2018) but it may detect, to some extent, undeclared species (Newmaster et al., 2013; Mezzasalma et al., 2017). DNA metabarcoding, the combination of high-throughput sequencing (HTS) and DNA barcoding, enables untargeted (Mezzasalma et al., 2017), simultaneous multi-taxa identification by using the DNA of different origins extracted from complex mixtures and matrices (Raclariu et al., 2018). Besides the indisputable analytical advantages brought to authentication, DNA barcoding and metabarcoding have also limits (Parveen et al., 2016; Raclariu et al., 2018) mainly due to the high sensitivity for any amplifiable DNA isolated from the product. One pollen grain from another species deposited on the harvested species can potentially lead to false-positives unless it originates from an allergenic or poisonous plant (Speranskaya et al., 2018; Mezzasalma et al., 2017) and then the method will became literally a lifesaver. False-negatives can be expected if the DNA has been degraded or lost during post-harvest processing or manufacturing unless it reports a falsely claimed herbal product which was illegally manufactured by mixing synthetic drugs and pharmaceuticals.

The use of DNA barcoding and metabarcoding for HPs’ authentication has allowed the detection of unlabeled species with allergenic potential, known or suspected toxicity, side-effects and/or negative interactions with other herbs, supplements or prescription medication which pose great risk for human health (Newmaster et al., 2013; Speranskaya et al., 2018). Furthermore, many species protected by the Convention on International Trade in Endangered Species of Wild Fauna and Flora (CITES) have been detected using DNA barcoding and metabarcoding in commercial HPs (Coghlan et al., 2015; de Boer et al., 2017), including DNA traces of the iconic snow leopard (*Panthera uncia*), a species with the highest level of trade restriction (Coghlan et al., 2015).

The scientific studies, reporting the authenticity of commercial herbal products using various DNA molecular diagnostic tools, have reported wide-ranging incongruences between the claimed and the identified species composition. The largest study to date has authenticated 1,436 commercial TCMs sold in China. Out of the 1,260 samples successfully DNA barcoded, 4.2% were adulterated (Han et al., 2016). When DNA barcoding was employed to test the authenticity of 44 herbal products sold in USA and Canada, 59% of the 40 products successfully analyzed contained DNA barcodes from plant species not listed on the labels (Newmaster et al., 2013). DNA barcoding coupled with next generation DNA sequencing (NGS) employed to authenticate 26 TCMs purchased within Australia has revealed that 13 samples contained DNA of undeclared plant or animal taxa from the total of 22 TCMs successfully analyzed (Coghlan et al., 2015). When Saint John’s wort (*Hypericum perforatum* L.) was an ingredient in 78 herbal products sold in 14 European countries, DNA metabarcoding has identified the species in only 68% of the 38 products successfully analyzed, but incongruence between constituent species and those listed on the label was detected in all products (Raclariu et al., 2017). Unfortunately, the scale of all the investigations is restrained to a specific geographical area, national or regional market, types of products, target plant species, product specific use or detection method (Ichim et al., 2018).

The reported values of adulterated commercial HPs are underestimated when classical DNA-based targeted approaches have been employed to mainly test the presence of labeled ingredient species, without having the technical capabilities to detect putative adulterant species. Our analysis illustrate that due to the ever-increasing analytical sensitivity of high throughput DNA sequencing the proportion of adulterated HPs is expected to significantly increase.

The problem of substandard and falsified medicinal products continues to increase, as globalized manufacturing and distribution systems grow ever more complex. Increasing demand, in addition to poor supply-chain management and the growth of e-commerce also creates opportunities for falsified medicines to be introduced into the supply chain (World Health Organization, 2018). This means that people are taking medicines that fail to treat or prevent disease but can cause serious illness or even death. Based on a WHO report of a 10% globally estimated presence of counterfeit medicinal products (World Health Organization, 2018), it was calculated that more than 200,000 persons may be dying each year from substandard and falsified antibiotics and antimalarials in Africa alone (World Health Organization, 2018). Unfortunately, many developing countries of Africa, parts of Asia and of Latin America have areas where more than 30% of the medicines on sale may be counterfeit (Newton et al., 2010). The health risks for the population increases considerably when approximately the same percentage of adulterated HPs is added for those consumers.

The herbal products contain more than one pharmacologically active ingredient and are often used in combination with conventional drugs. Adverse drug reactions (ADRs) (Jordan et al., 2010) due to herb–drug interactions (HDI) can appear in patients taking concomitantly herbs and prescribed medications (Awortwe et al., 2018). Several common herbal medicines interact with drugs, including St John’s wort (*H*. *perforatum* L.), ginkgo (*Ginkgo biloba* L.), ginger (*Zingiber officinale* Roscoe), ginseng (*Panax ginseng* C.A.Mey), green tea (*Camellia sinensis (L.) Kunze*), and garlic (*Allium sativum* L.) and are affecting the pharmacokinetic and pharmacodynamic properties of prescribed medications. On the other hand, the common drugs that interact with herbal medicines include warfarin, statins, midazolam, digoxin, amitriptyline, indinavir, cyclosporine, tacrolimus and irinotecan (Awortwe et al., 2018). Besides adulterant species, the herbal products were reported to contain many other harmful contaminants and residues, such as dust, pollen, insects, rodents, parasites, microbes, fungi, molds, mycotoxins, pesticides, PCBs, toxic heavy metals, radioactivity, processing impurities, solvent residues, illegal or prescription drugs (Boniglia et al., 2009; Jordan et al., 2010; Posadzki et al., 2013; Coghlan et al., 2015). The most severe adverse effects reported caused by the adulteration of herbal products were agranulocytosis, meningitis, multi-organ failure, perinatal stroke, arsenic, lead or mercury poisoning, malignancies or carcinomas, hepatic encephalopathy, hepatorenal syndrome, nephrotoxicity, rhabdomyolysis, metabolic acidosis, renal or liver failure, cerebral edema, coma, intracerebral hemorrhage, and death (Posadzki et al., 2013).

The global market of HPs plays already a significant role in the economic development of a number of countries (World Health Organization, 2013). Some countries have already seized the growth potential of this particular market as the herbal products are clearly gaining global influence in modern medical and health services. According to the WHO (World Health Organization, 2013), about 80% of world’s population relies on traditional medicine for their primary health care needs, and most of this therapy involves the use of plant extracts or their active components. The rapidly expanding global market of herbal products (Newmaster et al., 2013) is projected to reach US$ 115 billion by 2020 (Raclariu et al., 2018) *and the trade of medicinal plants* will continue to grow at the rate of 15–25% annually and will reach US$ 5 trillion by 2050 (Booker et al., 2012).

The increasing demand for herbals and the limited supply of many species that are harvested from the wild (Coghlan et al., 2015) is stimulating the economically-motivated adulteration (EMA) while the incidence of intentional adulterations is on the rise globally (Simmler et al., 2018). Reporting that as much as 27% of all commercial HPs are adulterated when tested with DNA-based methods alone, we confirm their worldwide spread and threat at consumers’ health and social security (Gao et al., 2017).

The HPs’ quality assurance and control are regulated primarily at the national level as a function of their legal status (Simmler et al., 2018). Due to differences and discrepancies between the national HPs’ regulatory frameworks (Mezzasalma et al., 2017; Raclariu et al., 2018), the same herbal product may be commercialized on different national markets either as food or medicine while is forbidden on others (Mezzasalma et al., 2017), all these with significant negative impact on HPs’ safety evaluation and pharmacovigilance (PV) (Coghlan et al., 2015; de Boer et al., 2015). The PV is essential for the development of reliable information on the safety of herbal products and relies on the product label information regarding the ingredients and the adherence to good manufacturing practices along the commercialization chain (de Boer et al., 2015). Authentication of constituents in herbal medicines using analytical chemistry methods can help detect contaminants and toxins, but are often limited or incapable of detecting the source of the contamination. Recent developments in molecular plant identification by using DNA sequence data enable accurate identification of plant species content in herbal medicines using defined DNA markers. DNA barcoding has the potential to be used as a standard method in herbal PV research of adverse reactions to specific products (de Boer et al., 2015).

This description of the current global situation should be useful for decision-makers to realize the considerable risks posed by adulterated HPs to human health as they are consumed for their claimed or expected benefits. The globalized HPs’ value chains are not supported by a harmonized framework for the evaluation of their quality and authenticity and this call for immediate action.

## Methods

### Databases

We systematically searched four databases (Web of Science, PubMed, Scopus, and ScienceDirect) for relevant, peer reviewed studies, using a combination of relevant keywords and Boolean operators “medicinal plant *OR* herbal product *OR* herbal medicine *OR* food supplement *OR* dietary supplement *OR* herbal supplement *OR* herbal remedy *OR* nutraceutical *OR* botanical *OR* herbal *OR* TCM *AND* DNA *OR* PCR *OR* barcode *OR* barcoding *OR* metabarcoding *AND* authentication *OR* authenticity *OR* authentic *OR* contamination *OR* contaminant *OR* substitution *OR* substitute *OR* filler”.

The database search was carried out for the period January 2000–December 2018, divided in three consecutive time periods: 2000–2016, 2017, and 2018– to allow a step-by-step analysis of the retrieved abstracts and full text publications. The option “search alert” has been activated for all the searches in the databases to receive weekly updates if other records are to be added, after the search was performed.

**Table:** 

Database	Period/year	No. of abstracts retrieved	No. of studies selected for full text reading
WoS	2000–2016	541	279
	2017	77	41
	2018–	88	50
			Duplication check
PubMed	2000–2016	471	59
	2017	58	14
	2018–	73	21
			Duplication check
Scopus	2000–2016	377	36
	2017	205	39
	2018–	47	5
			Duplication check
Science Direct	2000–2016	899	7
	2017	165	4
	2018^(*)^–	426	21
Total		3,427	576

^(*)^ScienceDirect has changed the “advanced search” form in 2018, e.g. by allowing maximum 8 Boolean operators. The following combination of keywords and Boolean operators has been used: (“medicinal plant” OR “herbal product” OR “food supplement”) AND (DNA OR PCR OR barcoding) AND (authentication OR contamination OR substitution), but the search was performed in all fields of the document (except the reference section), and not only in the title, abstract and keywords sections (as all the previous searches), to retrieve as many as possible articles to be further analyzed.

Furthermore, we used cross-referencing to identify additional peer-reviewed publications (irrespective of the publication year).

### Selection Criteria

Due to the very diverse reporting formats of the authenticity results, unifying criteria for selection had to be established and used for including the retrieved studies in our analysis:

1. The reported samples had to be “herbal products” *sensu lato*. The full broad spectrum of commercial names was searched for and accepted for being included in our analysis, such as herbal drugs, botanical drugs, botanicals, phytomedicines, traditional medicines (TMs), herbal medicines (HMs), traditional Chinese medicines (TCMs), traditional herbal medicines products (THMPs), natural health products (NHPs), dietary supplements (DSs), plant food supplements (PFSs) or food supplements (FSs) etc., all falling under two main categories: medicines or foods, with health benefits claims or only expected, respectively. For the plant species with multiple uses (*e.g.*, saffron is used both as traditional remedy and as a high-value spice) only studies referring to the medicinal properties of the herbal product or ingredient species were selected and included in our analysis.
2. The analyzed products had to be “commercial”. The following keywords were accepted: “purchased”, “bought”, and similar, no matter if the samples came from a local or traditional market, herbalist shop, health food store, supermarket, pharmacy, etc., purchased under prescription, freely over-the-counter (OTC) or *via* e-commerce. Our analysis excluded studies where the analyzed samples were “collected”, “procured” (with no other details given to establish their commercial value), obtained “cost-free”, a “gift” or “donated” by a person, institution or company. Furthermore, we excluded from our analysis the illegal/illicit products as well, such as the ones seized by Customs or Border Protection Services (or similar) or confiscated by the Police (or similar).
3. The products had to be clearly allocated to a “country” or “territory” (*e.g.*, Hong Kong). We have reported the country from which the products were purchased or received after they were ordered online (e-commerce), no matter which country they were imported or transported from. As such, we report the country where the consumer had the possibility to actually use a certain herbal product, and not the country where the herbal product was produced or imported from (frequently not specified by the authors or not even mentioned on the product’s label).
4. The conclusion “authentic”/”adulterated” (or similar) had to be drawn by the authors of the analyzed studies, or with their written assistance; in a few cases, (re)confirmation from the author(s) was sought by email correspondence, without disclosing the final purpose of the information requested, and kindly provided by the author(s). If their response/confirmation was obtained and selection criteria were met, the study was included in our analysis, but if there was no response or the selection criteria were not met, the respective study was excluded from our analysis. For all studies, our involvement was restricted to operations such as counting the samples, transforming percentages in absolute numbers, after having eliminated the samples from which DNA/sequences were not isolated/amplified/sequenced, without reinterpreting the experimental results (or similar) in any way.
5. The samples had to be analyzed with a “DNA-based method”, no matter which one. A wide variety of techniques were reviewed, starting from classical molecular markers (used in the early 2000) to DNA barcoding, and metabarcoding (in the recent years). When also other methods (*e.g.*, macro- and microscopic examination, phytochemical analysis) were used to test the authenticity of the (same) products, we took into consideration the DNA-based results only, as they were distinctively reported by authors.

The maps were created on mapchart.net

For each retrieved peer-reviewed, full text, study we examined its potential for meeting the selection criteria for inclusion in our analysis:

**Table:** 

Full text studies	Period/year			Total
	2000–2016	2017	2018–	
Full text studies selected from databases and subsequently analyzed	381	98	97	576
Full text studies identified by cross-referencing^(**)^ and subsequently analyzed	166	25	6	197
	Selection criteria applied			
Full text studies included in the analysis	129	41	36	206

^(^**^)^All the article suggestions received through the databases’ “alert” option were included in the cross-referencing category. Starting from 3,427 abstracts, we have analyzed 773 full text studies and, after applying our selection criteria, we included 206 peer-reviewed studies in our analysis.

## Author Contributions

The author confirms being the sole contributor of this work and approved it for publication.

## Funding

This work was supported by a grant from the Ministry of Research and Innovation through Program 1—Development of the National R&D System, Subprogram 1.2—Institutional Performance—Projects for Excellence Financing in RDI, contract no. 22PFE/2018. This publication was supported by the National Core Program funded by the Romanian Ministry of Research and Innovation, project number 25N/11.02.2019, BIODIVERS 19270401.

## Conflict of Interest

The author declares that the research was conducted in the absence of any commercial or financial relationships that could be construed as a potential conflict of interest.
