# Supplementary material for: The DNA-Based Authentication of Commercial Herbal Products Reveals Their Globally Widespread Adulteration
Source: Front Pharmacol. 2019 Oct 24;10:1227. doi: 10.3389/fphar.2019.01227 (PMC6822544; doi:10.3389/fphar.2019.01227)
Supplement: Supplementary file 1 [file DataSheet_1.pdf]

Supplementary Material

Table S1. The authenticity of commercial herbal products sold on national markets

No.	Continent	No.	Country	References
1.	Asia	1.	Bhutan	(29)
		2.	China	(12, 21, 29-106)
		3.	Hong Kong	(107)
		4.	India	(8, 108-130)
		5.	Iran	(19, 131)
		6.	Japan	(57, 74, 75, 132-144)
		7.	Malaysia	(145-148)
		8.	North Korea	(44)
		9.	Pakistan	(149, 150)

		10.	Philippines	(151-153)
		11.	Russia	(17)
		12.	Singapore	(31)
		13.	South Korea	(29, 45, 47, 48, 53-55, 66, 76, 154-157)
		14.	Taiwan	(39, 158-168)
		15.	Thailand	(6, 169-180)
		16.	United Arab Emirates	(181)
2	Europe	17.	Austria	(182-186)
		18.	Czech Republic	(22, 187)
		19.	France	(187)
		20.	Germany	(19, 22, 136, 188-191)
		21.	Greece	(19)
		22.	Italy	(182, 192, 193, 196)

		23.	Norway	(187)
		24.	Poland	(22, 183, 187)
		25.	Portugal	(197)
		26.	Romania	(22, 183, 187)
		27.	Spain	(22, 187)
		28.	Turkey	(19, 198)
		29.	United Kingdom	(22, 199-202)
3	Africa	30.	Morocco	(203)
		31.	South Africa	(204, 205)
		32.	Tanzania	(206)
4	North America	33.	Canada	(18, 207)
		34.	USA	(32, 50, 71, 134, 208-219)
5	South America	35.	Bolivia	(220)
		36.	Brazil	(221-224)

6	Australia	37.	Australia	(20, 225)

References (for SI reference citations):

29. Moon, B. C., Kim, W. J., Park, I., Sung, G. H. & Noh, P. Establishment of a PCR assay for the detection and discrimination of authentic Cordyceps and adulterant species in food and herbal medicines. *Molecules* **23,** 1932 (2018).

30. Zhang, W., Yang, S., Zhao, H. & Huang, L. Using the ITS2 sequence-structure as a DNA mini-barcode: A case study in authenticating the traditional medicine "Fang Feng". *Biochem. Syst. Ecol.* **69,** 188–194 (2016).

31. Cheng, X. *et al.* DNA extraction protocol for biological ingredient analysis of Liuwei Dihuang Wan. *Genomics. Proteomics Bioinformatics* **12,** 137–143 (2014).

32. Mihalov, J. J., Marderosian, A. D. & Pierce, J. C. DNA identification of commercial ginseng samples. *J. Agric. Food Chem.* **48,** 3744–52 (2000).

33. Liu Meizi. Authentication of Illicium verum using a DNA barcode psbA-trnH. *J. Med. Plants Res.* **6,** 3156–3161 (2012).

34. Zheng, C.-J., Zhao, S.-J., Zhao, Z.-H. & Guo, J. Molecular authentication of the traditional medicinal plant Fallopia multiflora. *Planta Med.* **75,** 870–872 (2009).

35. Yang, Y., Zhai, Y., Liu, T., Zhang, F. & Ji, Y. Detection of Valeriana jatamansi as an adulterant of medicinal Paris by length variation of chloroplast psb A -trn H region. *Planta Med.* **77,** 87–91 (2011).

36. Xin, T. *et al.* Precise species detection of traditional Chinese patent medicine by shotgun metagenomic sequencing. *Phytomedicine* **47,** 40–47 (2018).

37. Li, Q. *et al.* Quality control of the traditional Chinese medicine Ruyi jinhuang powder based on high-throughput sequencing and real-time PCR. *Sci. Rep.* **8,** 8261 (2018).

38. Chen, R. *et al.* DNA based identification of medicinal materials in Chinese patent medicines. *Sci. Rep.* **2,** 958 (2012).

39. Cao, H., But, P. P. & Shaw, P. C. Authentication of the Chinese drug 'ku-di-dan' (herba elephantopi) and its substitutes using random-primed polymerase chain reaction (PCR). *Acta Pharm. Sin.* **31,** 543–53 (1996).

40. Jia, J., Xu, Z., Xin, T., Shi, L. & Song, J. Quality control of the traditional patent medicine Yimu Wan based on SMRT sequencing and DNA barcoding. *Front. Plant Sci.* **8,** 926 (2017).

41. Chen, G. *et al.* Application of 3' Untranslated Region (UTR) sequence-based amplified polymorphism analysis in the rapid authentication of Radix astragali. *J. Agric. Food Chem.* **53,** 8551–8556 (2005).

42. Xue, C.-Y., Li, D.-Z. & Wang, Q.-Z. Application of LightCycler polymerase chain reaction and melting curve analysis to the authentication of the traditional Chinese medicinal plant Cimicifuga foetida. *Planta Med.* **75,** 873–875 (2009).

43. Wang, C.-Z., Li, P., Ding, J.-Y., Jin, G.-Q. & Yuan, C.-S. Identification of Fritillaria pallidiflora using diagnostic PCR and PCR-RFLP based on nuclear ribosomal DNA internal transcribed spacer sequences. *Planta Med.* **71,** 384–386 (2005).

44. Mizukami, H., Okabe, Y., Kohda, H. & Hiraoka, N. Identification of the crude drug atractylodes rhizome (Byaku-jutsu) and atractylodes lancea rhizome (So-jutsu) using chloroplast TrnK sequence as a molecular marker. *Biol. Pharm. Bull.* **23,** 589–594 (2000).

45. Kim, W., Yang, S., Choi, G. & Moon, B. Peptide nucleic acid based molecular authentication for identification of four medicinal Paeonia species using melting array analysis of the Internal Transcribed Spacer 2 region. *Molecules* **22,** 1922 (2017).

46. Law, S. K.-Y. *et al.* Molecular analyses of the Chinese herb Leigongteng (Tripterygium wilfordii Hook.f.). *Phytochemistry* **72,** 21–26 (2011).

47. Yang, J. Y., Kim, H.-K. & Park, S. J. Development of genetic marker specific for Korean hwanggi medicine (Radix Astragali). *Food Sci. Biotechnol.* **20,** 1561–1567 (2011).

48. Jigden, B., Wang, H., Samdan, N. & Yang, D.-C. Molecular identification of oriental medicinal plant Anemarrhena asphodeloides Bunge ('Jimo') by multiplex PCR. *Mol. Biol. Rep.* **37,** 955–960 (2010).

49. Zheng, C.-J., Zhao, S.-J. & Shao, L. Species identification of Chinese medicinal plant Fallopia multiflora (Thunb.) Haraldson by suppression subtraction hybridization. *J. Nat. Med.* **68,** 192–198 (2014).

50. Li, M. *et al.* Authentication of the anti-tumor herb Baihuasheshecao with bioactive marker compounds and molecular sequences. *Food Chem.* **119,** 1239–1245 (2010).

51. Jiang, C. *et al.* Barcoding melting curve analysis for rapid, sensitive, and discriminating authentication of saffron (Crocus sativus L.) from its adulterants. *Biomed Res. Int.* **2014,** 1–10 (2014).

52. Lu, C. *et al.* Development of randomly amplified polymorphic DNA-sequence characterized amplified region marker for identification of Apocynum venetum LINN. from A. pictum SCHRENK. *Biol. Pharm. Bull.* **33,** 522–6 (2010).

53. Noh, P., Kim, W. J., Yang, S., Park, I. & Moon, B. C. Authentication of the herbal medicine Angelicae Dahuricae Radix using an ITS sequence-based multiplex SCAR assay. *Molecules* **23,** (2018).

54. Kim, W. J. *et al.* Development of conventional PCR and real-time PCR assays to discriminate the origins of Chinese pepper oil and herbal materials from *Zanthoxylum*. *J. Sci. Food Agric.* (2018). doi:10.1002/jsfa.9458

55. Moon, B. *et al.* Differentiating authentic Adenophorae Radix from its adulterants in commercially-processed samples using multiplexed ITS sequence-based SCAR markers. *Appl. Sci.* **7,** 660 (2017).

56. Wei, Y., Chen, Y., Huang, Y., Liu, J. & Liang, Y. Molecular authentication and quantitative analysis of *Sarcandra glabra* and adulterated chloranthus products using SNP markers. *Mitochondrial DNA Part A* **27,** 3618–3625 (2016).

57. Yang, D.-Y., Fushimi, H., Cai, S.-Q. & Komatsu, K. Molecular analysis of Rheum species used as Rhei Rhizoma based on the chloroplast matK gene sequence and its application for identification. *Biol. Pharm. Bull.* **27,** 375–83 (2004).

58. Jiang, L.-L. *et al.* Helicase-dependent amplification is effective in distinguishing Asian ginseng from American ginseng. *Food Control* **43,** 199–205 (2014).

59. Cheng, X. *et al.* Biological ingredient analysis of traditional Chinese medicine preparation based on high-throughput sequencing: the story for Liuwei Dihuang Wan. *Sci. Rep.* **4,** 5147 (2015).

60. Zhao, S., Chen, X., Song, J., Pang, X. & Chen, S. Internal transcribed spacer 2 barcode: a good tool for identifying Acanthopanacis cortex. *Front. Plant Sci.* **6,** 840 (2015).

61. Wang, C. Z., Li, P., Ding, J. Y., Peng, X. & Yuan, C. S. Simultaneous identification of Bulbus Fritillariae cirrhosae using PCR-RFLP analysis. *Phytomedicine* **14,** 628–632 (2007).

62. Xin, G.-Z. *et al.* Authentication of Bulbus Fritillariae Cirrhosae by RAPD-derived DNA markers. *Molecules* **19,** 3450–3459 (2014).

63. Yu, X., Xie, Z., Wu, J., Tao, J. & Xu, X. DNA barcoding identification of kadsurae caulis and spatholobi caulis based on internal transcribed spacer 2 region and secondary structure prediction. *Pharmacogn. Mag.* **12,** 165 (2016).

64. Li, M. *et al.* Identification of Baiying (Herba Solani Lyrati) commodity and its toxic substitute Xungufeng (Herba Aristolochiae Mollissimae) using DNA barcoding and chemical profiling techniques. *Food Chem.* **135,** 1653–1658 (2012).

65. Liu, Y. *et al.* DNA barcoding based identification of Hippophae species and authentication of commercial products by high resolution melting analysis. *Food Chem.* **242,** 62–67 (2018).

66. Kim, W. *et al.* Rapid authentication of the herbal medicine plant species Aralia continentalis Kitag. and Angelica biserrata C.Q. Yuan and R.H. Shan using ITS2 sequences and multiplex-SCAR markers. *Molecules* **21,** 270 (2016).

67. Duan, B.-Z. *et al.* Authenticity analyses of Rhizoma Paridis using barcoding coupled with high

resolution melting (Bar-HRM) analysis to control its quality for medicinal plant product. *Chin. Med.* **13,** 8 (2018).

68. Xin, T. *et al.* Biomonitoring for traditional herbal medicinal products using DNA metabarcoding and single molecule, real-time sequencing. *Acta Pharm. Sin. B* **8,** 488–497 (2018).

69. Huang, W.-J., Li, F.-F., Liu, Y.-J. & Long, C.-L. Identification of Crocus sativus and its adulterants from Chinese markets by using DNA barcoding technique. *Iran. J. Biotechnol.* **13,** 36–42 (2015).

70. Guo, X., Wang, X., Su, W., Zhang, G. & Zhou, R. DNA barcodes for discriminating the medicinal plant Scutellaria baicalensis (Lamiaceae) and its adulterants. *Biol. Pharm. Bull.* **34,** 1198–203 (2011).

71. Harnly, J. *et al.* Comparison of flow injection MS, NMR, and DNA sequencing: Methods for identification and authentication of black cohosh (Actaea racemosa). *Planta Med.* **82,** 250–262 (2015).

72. Fang, W. *et al.* Identification of the varietal origin of processed loose-leaf tea based on analysis of a single leaf by SNP nanofluidic array. *Crop J.* **4,** 304–312 (2016).

73. Da, G. *et al.* Identification of Ardisiae Japonicae Herba by ITS2 sequence. *J. Chinese Med. Mater.* **38,** 2277–80 (2015).

74. Sasaki, Y., Fushimi, H., Cao, H., Cai, S.-Q. & Komatsu, K. Sequence analysis of Chinese and Japanese Curcuma drugs on the 18S rRNA gene and trnK gene and the application of amplification-refractory mutation system analysis for their authentication. *Biol. Pharm. Bull.* **25,** 1593–9 (2002).

75. Maruyama, T. *et al.* Authentication of the traditional medicinal plant Eleutherococcus senticosus by DNA and chemical analyses. *Planta Med.* **74,** 787–789 (2008).

76. Sato-Masumoto, N. *et al.* Survey on the original plant species of crude drugs distributed as Cynanchi Wilfordii Radix and its related crude drugs in the Korean and Chinese markets. *Biol.*

Pharm. Bull. **40,** 1693–1699 (2017).

77. Hai-bo, H. *et al.* Identification of Geranii Herbae using ITS2 sequence. *Chinese Pharm. J.* 1505–1511 (2015). doi:10.11669/cpj.2015.17.010

78. Yang, D.-Y., Fushimi, H., Cai, S.-Q. & Komatsu, K. Polymerase chain reaction-restriction fragment length polymorphism (PCR-RFLP) and amplification refractory mutation system (ARMS) analyses of medicinally used Rheum species and their application for identification of Rhei Rhizoma. *Biol. Pharm. Bull.* **27,** 661–9 (2004).

79. Jiang, C. *et al.* Conventional octaplex PCR for the simultaneous identification of eight mainstream closely related Dendrobium species. *Ind. Crops Prod.* **112,** 569–576 (2018).

80. Sun, W. *et al.* Study of commercially available Lobelia chinensis products using Bar-HRM technology. *Front. Plant Sci.* **8,** 351 (2017).

81. Wu, L. *et al.* An integrated system for identifying the hidden assassins in traditional medicines containing aristolochic acids. *Sci. Rep.* **5,** 11318 (2015).

82. Chen X.-L, Xiang L, Mei, Q.-X. Identification of Rubi Parvifolii radix and its adulterants using DNA barcoding. *Chinese Pharm. J.* **50,** 1490–1495 (2015).

83. Xin, T. *et al.* Super food Lycium barbarum (Solanaceae) traceability via an internal transcribed spacer 2 barcode. *Food Res. Int.* **54,** 1699–1704 (2013).

84. Zhou, H. *et al.* QR code labeling system for Xueteng-related herbs based on DNA barcode. *Chinese Herb. Med.* (2018). doi:10.1016/J.CHMED.2018.09.006

85. Duan, B.-Z. *et al.* Survey of traditional Dai medicine reveals species confusion and potential safety concerns: a case study on Radix Clerodendri Japonicum. *Chin. J. Nat. Med.* **15,** 417–426 (2017).

86. Ma, X.-C., Yao, H., Xin, T., Chen, X.-C. & Song, J.-Y. SNP identification of Alisma orientale, Alisma plantago-aquatica and Alismatis Rhizoma in the market based on DNA barcoding. *Chinese Pharm. J.* **50,** 1474–1478 (2015).

87. Shi, Y. *et al.* Rapidly discriminate commercial medicinal Pulsatilla chinensis (Bge.) Regel

from its adulterants using ITS2 barcoding and specific PCR-RFLP assay. *Sci. Rep.* **7,** 40000 (2017).

88. Lu, S. *et al.* Confirming the genetic identity of Dendrobium fimbriatum using an Amplification Refractory Mutation System (ARMS). *Plant Mol. Biol. Report.* **28,** 712–716 (2010).

89. Yuan, Q.-J. *et al.* Identification of species and *materia medica* within *Angelica* L. (Umbelliferae) based on phylogeny inferred from DNA barcodes. *Mol. Ecol. Resour.* **15,** 358–371 (2015).

90. Gao, Z., Wang, L., Wang, X., Liu, Y. & Han, J. Authenticity survey of Cuscutae Semen on markets using DNA barcoding. *Chinese Herb. Med.* **9,** 218–225 (2017).

91. Liu, Y., Wang, X., Wei, X., Gao, Z. & Han, J. Rapid authentication of Ginkgo biloba herbal products using the Recombinase Polymerase Amplification assay. *Sci. Rep.* **8,** 8002 (2018).

92. Guo, Y. *et al.* DNA authentication of So-jutsu (Atractylodes lancea rhizome) and Byaku-jutsu (Atractylodes rhizome) obtained in the market based on the nucleotide sequence of the 18S–5.8S rDNA internal transcribed spacer region. *J. Nat. Med.* **60,** 149–156 (2006).

93. Zhu, X., Zhang, Y., Liu, X., Hou, D. & Gao, T. Authentication of commercial processed Glehniae Radix (Beishashen) by DNA barcodes. *Chin. Med.* **10,** 35 (2015).

94. Long, P. *et al.* Commercialized non-Camellia tea: traditional function and molecular identification. *Acta Pharm. Sin. B* **4,** 227–237 (2014).

95. Yu, N. *et al.* Suitable DNA barcoding for identification and supervision of Piper kadsura in Chinese medicine markets. *Molecules* **21,** 1221 (2016).

96. Wang, X., Liu, Y., Wang, L., Han, J. & Chen, S. A nucleotide signature for the identification of Angelicae Sinensis Radix (Danggui) and its products. *Sci. Rep.* **6,** 34940 (2016).

97. Zhang, J. *et al.* Investigation on species authenticity for herbal products of Celastrus Orbiculatus and Tripterygum Wilfordii from markets using ITS2 barcoding. *Molecules* **23,** 967 (2018).

98. Li, J. *et al.* Using SSR-HRM to identify closely related species in herbal medicine products: A case study on licorice. *Front. Pharmacol.* **9,** 407 (2018).

99. Ding, G. *et al.* SNP, ARMS and SSH authentication of medicinal Dendrobium officinale KIMURA et MIGO and application for identification of Fengdou drugs. *Biol. Pharm. Bull.* **31,** 553–7 (2008).

100. Song, M., Li, J., Xiong, C., Liu, H. & Liang, J. Applying high-resolution melting (HRM) technology to identify five commonly used Artemisia species. *Sci. Rep.* **6,** 34133 (2016).

101. Yu, N. *et al.* Integrated approach for identifying and evaluating the quality of Marsdenia tenacissima in the medicine market. *PLoS One* **13,** e0195240 (2018).

102. Guo, M., Ren, L. & Pang, X. Inspecting the true identity of herbal materials from Cynanchum using ITS2 barcode. *Front. Plant Sci.* **8,** 1945 (2017).

103. Wang, X.-Y. *et al.* Detection of Cistanches Herba (Rou Cong Rong) medicinal products using species-specific nucleotide signatures. *Front. Plant Sci.* **9,** 1643 (2018).

104. Xin, T. *et al.* Survey of commercial Rhodiola products revealed species diversity and potential safety issues. *Sci. Rep.* **5,** 8337 (2015).

105. Jiang, C. *et al.* Fluorescence visual detection of herbal product substitutions at terminal herbal markets by CCP-based FRET technique. *Sci. Rep.* **6,** 35540 (2016).

106. Xiong, C. *et al.* Identifying the species of seeds in traditional Chinese medicine using DNA barcoding. *Front. Pharmacol.* **9,** 701 (2018).

107. Lo, Y.-T., Li, M. & Shaw, P.-C. Identification of constituent herbs in ginseng decoctions by DNA markers. *Chin. Med.* **10,** 1 (2015).

108. Frommenwiler, D. A. *et al.* Qualitative and quantitative characterization of two licorice root species (Glycyrrhiza glabra L. and Glycyrrhiza uralensis Fisch.) by HPTLC, validated by HPLC and DNA sequencing. *JPC - J. Planar Chromatogr. - Mod. TLC* **30,** 467–473 (2017).

109. Sasikumar, B., Syamkumar, S., Remya, R. & John Zachariah, T. PCR based detection of adulteration in the market samples of turmeric powder. *Food Biotechnol.* **18,** 299–306 (2004).

110. Irshad, S., Singh, J., Kakkar, P. & Mehrotra, S. Molecular characterization of Desmodium species — An important ingredient of ‘Dashmoola’ by RAPD analysis. *Fitoterapia* **80,** 115–118 (2009).

111. Seethapathy, G. S., Balasubramani, S. P. & Venkatasubramanian, P. nrDNA ITS sequence based SCAR marker to authenticate Aconitum heterophyllum and Cyperus rotundus in Ayurvedic raw drug source and prepared herbal products. *Food Chem.* **145,** 1015–1020 (2014).

112. Kumbhalkar, B., Upadhye, A. & Tamhankar, S. Molecular authentication of Trichosanthes species traded as “Patola:” An ayurvedic drug resource. *Pharmacogn. Mag.* **14,** 52 (2018).

113. Dnyaneshwar, W., Preeti, C., Kalpana, J. & Bhushan, P. Development and application of RAPD-SCAR marker for identification of Phyllanthus emblica LINN. *Biol. Pharm. Bull.* **29,** 2313–6 (2006).

114. Tamhankar, S., Ghate, V., Raut, A. & Rajput, B. Molecular profiling of “Chirayat” complex using Inter Simple Sequence Repeat (ISSR) markers. *Planta Med.* **75,** 1266–1270 (2009).

115. Swetha, V. P., Parvathy, V. A., Sheeja, T. E. & Sasikumar, B. Authentication of Myristica fragrans Houtt. using DNA barcoding. *Food Control* **73,** 1010–1015 (2017).

116. Ganie, S. H., Srivastava, P. S., Narula, A., Ali, Z. & Sharma, M. P. Authentication of shankhpushpi by RAPD markers. *EurAsian J. Biosci.* **6,** 39–46 (2012).

117. Yadav, A. Development of Sequence Characterized Amplified Region (SCAR) marker for the authentication of Bacopa monnieri (L.) Wettst. *European J. Med. Plants* **2,** 186–198 (2012).

118. Santhosh Kumar, J. U. *et al.* DNA barcoding to assess species adulteration in raw drug trade of “Bala” (genus: Sida L.) herbal products in South India. *Biochem. Syst. Ecol.* **61,** 501–509 (2015).

119. Mishra, P., Shukla, A. K. & Sundaresan, V. Candidate DNA barcode tags combined with High Resolution Melting (Bar-HRM) curve analysis for authentication of Senna alexandrina Mill. with validation in crude drugs. *Front. Plant Sci.* **9,** 283 (2018).

120. Srirama, R. *et al.* Assessing species admixtures in raw drug trade of Phyllanthus, a hepato-protective plant using molecular tools. *J. Ethnopharmacol.* **130,** 208–215 (2010).

121. Parvathy, V. A., Swetha, V. P., Sheeja, T. E. & Sasikumar, B. Detection of plant-based adulterants in turmeric powder using DNA barcoding. *Pharm. Biol.* **53,** 1774–1779 (2015).

122. Arunraj, R., Pasupathi, R., Abiramavalli, M. & Rathinasabapathi, P. DNA barcoding identifies the component species in the powder formulations of plant derived raw drugs sold in retail market in India. *Res. J. Biotechnol.* **11,** 1–8 (2016).

123. Malik, S., Priya, A. & Babbar, S. B. Employing barcoding markers to authenticate selected endangered medicinal plants traded in Indian markets. *Physiol. Mol. Biol. Plants* 1–11 (2018). doi:10.1007/s12298-018-0610-8

124. Seethapathy, G. S. *et al.* Authentication of Garcinia fruits and food supplements using DNA barcoding and NMR spectroscopy. *Sci. Rep.* **8,** 10561 (2018).

125. Vassou, S. L., Kusuma, G. & Parani, M. DNA barcoding for species identification from dried and powdered plant parts: A case study with authentication of the raw drug market samples of Sida cordifolia. *Gene* **559,** 86–93 (2015).

126. Urumarudappa, S. K. J. *et al.* DNA barcoding and NMR spectroscopy-based assessment of species adulteration in the raw herbal trade of Saraca asoca (Roxb.) Willd, an important medicinal plant. *Int. J. Legal Med.* **130,** 1457–1470 (2016).

127. Seethapathy, G. S. *et al.* Assessing product adulteration in natural health products for laxative yielding plants, Cassia, Senna, and Chamaecrista, in Southern India using DNA barcoding. *Int. J. Legal Med.* **129,** 693–700 (2015).

128. Vassou, S. L., Nithaniyal, S., Raju, B. & Parani, M. Creation of reference DNA barcode library and authentication of medicinal plant raw drugs used in Ayurvedic medicine. *BMC Complement. Altern. Med.* **16 Suppl 1,** 186 (2016).

129. Nithaniyal, S., Vassou, S. L., Poovitha, S., Raju, B. & Parani, M. Identification of species adulteration in traded medicinal plant raw drugs using DNA barcoding. *Genome* **60,** 139–146 (2017).

130. Santhosh Kumar, J. U. *et al.* Assessment of adulteration in raw herbal trade of important medicinal plants of India using DNA barcoding. *3 Biotech* **8,** 135 (2018).

131. Ghorbani, A., Saeedi, Y. & de Boer, H. J. Unidentifiable by morphology: DNA barcoding of plant material in local markets in Iran. *PLoS One* **12,** e0175722 (2017).

132. Kikuchi, H., Uchiyama, N., Ogata, J., Kikura-Hanajiri, R. & Goda, Y. Chemical constituents and DNA sequence analysis of a psychotropic herbal product. *Forensic Toxicol.* **28,** 77–83 (2010).

133. Fushimi, H., Komatsu, K., Isobe, M. & Namba, T. Application of PCR-RFLP and MASA Analyses on 18S Ribosomal RNA gene sequence for the identification of three ginseng drugs. *Biol. Pharm. Bull.* **20,** 765–769 (1997).

134. Zhu, S., Fushimi, H. & Komatsu, K. Development of a DNA microarray for authentication of ginseng drugs based on 18S rRNA gene sequence. *J. Agric. Food Chem.* **56,** 3953–3959 (2008).

135. Maruyama, T. *et al.* Authentication and chemical study of isodonis herba and isodonis extracts. *Chem. Pharm. Bull. (Tokyo).* **55,** 1626–30 (2007).

136. Oshima, N. *et al.* Identification of new diterpenes as putative marker compounds distinguishing Agnus Castus fruit (Chaste tree) from shrub Chaste tree fruit (Viticis Fructus). *Planta Med.* **82,** 147–153 (2016).

137. Masada-Atsumi, S. *et al.* Genome-based authentication of black cohosh (Cimicifuga racemosa; Ranunculaceae) supplements available in the Japanese markets. *Japanese J. Food Chem. Saf.* **20,** 178–189 (2013).

138. Masada, S. Authentication of the botanical origin of Western herbal products using Cimicifuga and Vitex products as examples. *J. Nat. Med.* **70,** 361–375 (2016).

139. Hiroyuki, K., Maruyama, T., Sugimura, K., Iida, O. & Goda, Y. The source plants and the chemical composition of the passion flower products available as dietary supplements. *Japanese J. Food Chem. Saf.* **17,** 198–206 (2010).

140. Maruyama, T., Kawamura, M., Kikura-Hanajiri, R., Takayama, H. & Goda, Y. The botanical origin of kratom (Mitragyna speciosa; Rubiaceae) available as abused drugs in the Japanese markets. *J. Nat. Med.* **63,** 340–344 (2009).

141. Yukie Kumeta, Takuro Maruyama, Daigo Wakana, Hiroyuki Kamakura, Y. G. Method for identifying the botanical origin of shatavari products and its application for survey analysis of products in the Japanese market. *Japanese J. Food Chem. Saf.* **18,** 163–167 (2011).

142. Masada-Atsumi, S., Kumeta, Y., Takahashi, Y., Hakamatsuka, T. & Goda, Y. Evaluation of the botanical origin of black cohosh products by genetic and chemical analyses. *Biol. Pharm. Bull.* **37,** 454–60 (2014).

143. Guo, Y. *et al.* Sequence analysis of chloroplast chlB gene of medicinal Ephedra species and its application to authentication of Ephedra herb. *Biol. Pharm. Bull.* **29,** 1207–11 (2006).

144. Zhu, S. *et al.* Genetic and chemical diversity of Eleutherococcus senticosus and molecular identification of Siberian ginseng by PCR-RFLP analysis based on chloroplast trnK intron sequence. *Food Chem.* **129,** 1844–1850 (2011).

145. Abubakar, B. M., Salleh, F. M., Shamsir Omar, M. S. & Wagiran, A. Assessing product adulteration of *Eurycoma longifolia* (Tongkat Ali) herbal medicinal product using DNA barcoding and HPLC analysis. *Pharm. Biol.* **56,** 368–377 (2018).

146. Fadzil, N., Wagiran, A., Mohd Salleh, F., Abdullah, S. & Mohd Izham, N. Authenticity testing and detection of Eurycoma longifolia in commercial herbal products using Bar-High Resolution Melting analysis. *Genes (Basel).* **9,** 408 (2018).

147. Tnah, L. H. *et al.* DNA barcode database of common herbal plants in the tropics: a resource for herbal product authentication. *Food Control* **95,** 318–326 (2019).

148. Asing, A. E. *et al.* Duplex real-time PCR assay using SYBR Green to detect and quantify Malayan box turtle (Cuora amboinensis) materials in meatballs, burgers, frankfurters and traditional Chinese herbal jelly powder. *Food Addit. Contam. Part A* **33,** 1643–1659 (2016).

149. Butt, J. *et al.* Authentication of polyherbal formulations using PCR technique. *Int. J. Pharmacogn.* **5,** 382–390 (2018).

150. Zahra, N. B., Shinwari, Z. K. & Qaiser, M. DNA barcoding: a tool for standardization of herbal medicinal products (HMPs) of Lamiaceae from Pakistan. *Pakistan J. Bot.* **48,** 2167–2174 (2016).

151. Olivar, J. E. C. *et al.* Establishment of a standard reference material (SRM) herbal DNA barcode library of *Vitex negundo* L. (lagundi) for quality control measures. *Food Addit. Contam. Part A* **33,** 741–748 (2016).

152. Raterta, R., Louie Cabelin, V. D. & Jonathan Alejandro, G. D. Molecular authentication of selected commercially sold medicinal plants in Quiapo, Manila, Philippines. *Int. J. Sci. Technol. Res.* **3,** 22–26 (2014).

153. Pedales, R. D. *et al.* DNA barcoding of Philippine herbal medicinal products. *J. AOAC Int.* **99,** 1479–1489 (2016).

154. Wang, H. *et al.* Molecular authentication of the oriental medicines Pericarpium Citri Reticulatae and Citri Unshius Pericarpium using SNP markers. *Gene* **494,** 92–95 (2012).

155. Choo, B. K. *et al.* Development of SCAR markers for the discrimination of three species of medicinal plants, Angelica decursiva (Peucedanum decursivum), Peucedanum praeruptorum and Anthricus sylvestris, based on the internal transcribed spacer (ITS) sequence and RAPD. *Biol. Pharm. Bull.* **32,** 24–30 (2009).

156. Liu, J. *et al.* BOKP: A DNA barcode reference library for monitoring herbal drugs in the Korean Pharmacopeia. *Front. Pharmacol.* **8,** 931 (2017).

157. Kim, Y. *et al.* Molecular discrimination of Cynanchum wilfordii and Cynanchum auriculatum by InDel markers of chloroplast DNA. *Molecules* **23,** 1337 (2018).

158. Chen, K. T. *et al.* Identification of Atractylodes plants in Chinese herbs and formulations by random amplified polymorphic DNA. *Acta Pharmacol. Sin.* **22,** 493–7 (2001).

159. Tsu-Hwie, L., Hong-Mou, L. & Rey-Yuh, W. Identification of Astragalus medicines using SCAR markers. *J. Food Drug Anal.* **16,** 57–62 (2008).

160. Lin, T. C., Yeh, M. S., Cheng, Y. M., Lin, L. C. & Sung, J. M. Using ITS2 PCR-RFLP to

generate molecular markers for authentication of Sophora flavescens Ait. *J. Sci. Food Agric.* **92,** 892–8 (2012).

161. Kuo, H.-C., Su, Y.-L., Yang, H.-L., Huang, I.-C. & Chen, T.-Y. Differentiation of Cordyceps Sinensis by a PCR-Single-Stranded Conformation Polymorphism-based method and characterization of the fermented products in Taiwan. *Food Biotechnol.* **20,** 161–170 (2006).

162. Lu, K.-T., Cheng, H.-Y., Lo, C.-F., Chang, H.-C. & Lin, J.-H. Discriminating between Achyranthis Bidentatae Radix and Cyathulae Radix in Chinese medicine preparations by Nested PCR and DNA sequencing methods. *Planta Med.* **73,** 1322–1326 (2007).

163. Lu, K.-T., Hsieh, Y.-C., Lee, H.-C., Liu, Y.-C. & Lo, C.-F. Authentication of Gardeniae Fructus in Chinese medicine preparations by Nested PCR and DNA sequencing methods. *J. Food Drug Anal.* **20,** 974–977 (2012).

164. Kang-Tsu, L., Hui-Chun, L., Fang-Su Liu, Chi-Fang, L. & Jer-Huei, L. Discriminating Astragali Radix from Hedysarum Radix in Chinese medicine preparations using Nested PCR and DNA sequencing methods. *J. Food Drug Anal.* **17,** 380–385 (2009).

165. Lu, K.-T., Lo, C.-F., Chang, H.-C. & Lin, J.-H. Identification of Saposhinkoviae Radix in concentrated Chinese medicine preparations by Nested PCR and DNA sequencing methods. *J. Food Drug Anal.* **13,** 219–224 (2005).

166. Lu, K.-T., Lee, H.-C., Liu, F.-S., Lo, C.-F. & Lin, J.-H. Identification of Ginseng Radix in Chinese medicine preparations by Nested PCR-DNA sequencing method and Nested PCR-Restriction Fragment Length Polymorphism. *J. Food Drug Anal.* **18,** 58–63 (2010).

167. Lee, S.-C., Wang, C.-H., Yen, C.-E. & Chang, C. DNA barcode and identification of the varieties and provenances of Taiwan's domestic and imported made teas using ribosomal internal transcribed spacer 2 sequences. *J. Food Drug Anal.* **25,** 260–274 (2017).

168. Lu, K.-T. Identification of botanical origin in Chinese medicinal preparations by Nested PCR and DNA sequencing methods. *J. Food Drug Anal.* **20,** 786–793 (2012).

169. Osathanunkul, M., Osathanunkul, K., Wongwanakul, S., Osathanunkul, R. & Madesis, P. Multiuse of Bar-HRM for Ophiocordyceps sinensis identification and authentication. *Sci. Rep.*

8, 12770 (2018).

170. Tungphatthong, C. *et al.* DNA barcoding of species of *Bacopa* coupled with high-resolution melting analysis. *Genome* 1–11 (2018). doi:10.1139/gen-2018-0059

171. Buddhachat, K., Osathanunkul, M., Madesis, P., Chomdej, S. & Ongchai, S. Authenticity analyses of Phyllanthus amarus using barcoding coupled with HRM analysis to control its quality for medicinal plant product. *Gene* **573,** 84–90 (2015).

172. Dechbumroong, P., Aumnouypol, S., Denduangboripant, J. & Sukrong, S. DNA barcoding of Aristolochia plants and development of species-specific multiplex PCR to aid HPTLC in ascertainment of Aristolochia herbal materials. *PLoS One* **13,** e0202625 (2018).

173. Wiriyakarun, S. *et al.* Discrimination of the Thai rejuvenating herbs Pueraria candollei (White Kwao Khruea), Butea superba (Red Kwao Khruea), and Mucuna collettii (Black Kwao Khruea) using PCR-RFLP. *J. Nat. Med.* **67,** 562–570 (2013).

174. Singtonat, S. & Osathanunkul, M. Fast and reliable detection of toxic Crotalaria spectabilis Roth. in Thunbergia laurifolia Lindl. herbal products using DNA barcoding coupled with HRM analysis. *BMC Complement. Altern. Med.* **15,** 162 (2015).

175. Boonsom, T., Waranuch, N., Ingkaninan, K., Denduangboripant, J. & Sukrong, S. Molecular analysis of the genus Asparagus based on matK sequences and its application to identify A. racemosus, a medicinally phytoestrogenic species. *Fitoterapia* **83,** 947–953 (2012).

176. Osathanunkul, M. *et al.* Hybrid analysis (barcode-high resolution melting) for authentication of Thai herbal products, Andrographis paniculata (Burm.f.) Wall.ex Nees. *Pharmacogn. Mag.* **12,** 71 (2016).

177. Osathanunkul, M. Bar-HRM for authenticating soursop (Annona muricata) tea. *Sci. Rep.* **8,** 12666 (2018).

178. Manissorn, J., Sukrong, S., Ruangrungsi, N. & Mizukami, H. Molecular phylogenetic analysis of Phyllanthus species in Thailand and the application of polymerase chain reaction-restriction fragment length polymorphism for Phyllanthus amarus identification. *Biol. Pharm. Bull.* **33,**

1723–7 (2010).

179. Intharuksa, A. *et al.* Molecular analysis of Terminalia spp. distributed in Thailand and authentication of crude drugs from Terminalia plants. *Biol. Pharm. Bull.* **39,** 492–501 (2016).

180. Osathanunkul, M., Madesis, P. & de Boer, H. Bar-HRM for authentication of plant-based medicines: Evaluation of three medicinal products derived from Acanthaceae species. *PLoS One* **10,** e0128476 (2015).

181. Mosa, K. A. *et al.* Using DNA barcoding to detect adulteration in different herbal plant-based products in the United Arab Emirates: Proof of concept and validation. *Recent Pat. Food. Nutr. Agric.* **9,** 55–64 (2018).

182. Ruzicka, J., Schmiderer, C. & Novak, J. DNA-based identification of Valeriana officinalis s.l.: a multiplexed amplification Refractory Mutation System (MARMS) and High Resolution Melting Curve Analysis (HRMA). *Plant Mol. Biol. Report.* **34,** 909–922 (2016).

183. Raclariu, A. C. *et al.* Veronica officinalis product authentication using DNA metabarcoding and HPLC-MS reveals widespread adulteration with Veronica chamaedrys. *Front. Pharmacol.* **8,** 378 (2017).

184. Ruzicka, J. *et al.* Identification of Verbena officinalis based on ITS sequence analysis and RAPD-derived molecular markers. *Planta Med.* **75,** 1271–1276 (2009).

185. Novak, J., Grausgruber-Gröger, S. & Lukas, B. DNA-based authentication of plant extracts. *Food Res. Int.* **40,** 388–392 (2007).

186. Schmiderer, C., Ruzicka, J. & Novak, J. DNA-based identification of Peucedanum ostruthium specimens and detection of common adulterants by high-resolution melting curve analysis. *Mol. Cell. Probes* **29,** 343–350 (2015).

187. Raclariu, A. C. *et al.* What's in the box? Authentication of Echinacea herbal products using DNA metabarcoding and HPTLC. *Phytomedicine* **44,** 32–38 (2018).

188. Doganay-Knapp, K., Orland, A., König, G. M. & Knöss, W. The potential of three different PCR-related approaches for the authentication of mixtures of herbal substances and finished

herbal medicinal products. *Phytomedicine* **43,** 60–67 (2018).

189. Horn, T. *et al.* Genetic authentication by RFLP versus ARMS? The case of Moldavian dragonhead (Dracocephalum moldavica L.). *Eur. Food Res. Technol.* **238,** 93–104 (2014).

190. Horn, T. & Häser, A. Bamboo tea: reduction of taxonomic complexity and application of DNA diagnostics based on *rbcL* and *matK* sequence data. *PeerJ* **4,** e2781 (2016).

191. Jürges, G. *et al.* Product authenticity versus globalisation—The Tulsi case. *PLoS One* **13,** e0207763 (2018).

192. Torelli, A., Marieschi, M. & Bruni, R. Authentication of saffron (Crocus sativus L.) in different processed, retail products by means of SCAR markers. *Food Control* **36,** 126–131 (2014).

193. Cornara, L. *et al.* Zornia latifolia: a smart drug being adulterated by Stylosanthes guianensis. *Int. J. Legal Med.* **132,** 1321–1331 (2018).

194. Marieschi, M., Torelli, A., Beghé, D. & Bruni, R. Authentication of Punica granatum L.: Development of SCAR markers for the detection of 10 fruits potentially used in economically motivated adulteration. *Food Chem.* **202,** 438–444 (2016).

195. Serrone, P. Del, Attorri, L. & Palazzino, G. Easy DNA extraction for rapid detection of *Panax ginseng* C. A. Meyer in commercial ginseng products. *Nat. Prod. Res.* **21,** 1099–1103 (2007).

196. De Castro, O. *et al.* What is in your cup of tea? DNA Verity Test to characterize black and green commercial teas. *PLoS One* **12,** e0178262 (2017).

197. Costa, J. *et al.* HRM analysis targeting ITS1 and matK loci as potential DNA mini-barcodes for the authentication of Hypericum perforatum and Hypericum androsaemum in herbal infusions. *Food Control* **61,** 105–114 (2016).

198. Uncu, A. T., Uncu, A. O., Frary, A. & Doganlar, S. Authentication of botanical origin in herbal teas by plastid noncoding DNA length polymorphisms. *J. Agric. Food Chem.* **63,** 5920–5929 (2015).

199. Howard, C. *et al.* PlantID - DNA-based identification of multiple medicinal plants in complex mixtures. *Chin. Med.* **7,** 18 (2012).

200. Howard, C. *et al.* Molecular identification of Hypericum perforatum by PCR amplification of the ITS and 5.8S rDNA Region. *Planta Med.* **75,** 864–869 (2009).

201. Kazi, T., Hussain, N., Bremner, P., Slater, A. & Howard, C. The application of a DNA-based identification technique to over-the-counter herbal medicines. *Fitoterapia* **87,** 27–30 (2013).

202. Ruhsam, M. & Hollingsworth, P. M. Authentication of Eleutherococcus and Rhodiola herbal supplement products in the United Kingdom. *J. Pharm. Biomed. Anal.* **149,** 403–409 (2018).

203. Kool, A. *et al.* Molecular identification of commercialized medicinal plants in southern Morocco. *PLoS One* **7,** e39459 (2012).

204. Pretorius, E., Van der Bank, M. & Viljoen, A. M. DNA Barcoding detects contamination and substitution in herbal products containing Harpagophytum spp. *South African J. Bot.* **109,** 364–365 (2017).

205. Mankga, L. T., Yessoufou, K., Moteetee, A. M., Daru, B. H. & van der Bank, M. Efficacy of the core DNA barcodes in identifying processed and poorly conserved plant materials commonly used in South African traditional medicine. *Zookeys* 215–33 (2013). doi:10.3897/zookeys.365.5730

206. Veldman, S. *et al.* High-throughput sequencing of African chikanda cake highlights conservation challenges in orchids. *Biodivers. Conserv.* **26,** 2029–2046 (2017).

207. Ivanova, N. V., Kuzmina, M. L., Braukmann, T. W. A., Borisenko, A. V. & Zakharov, E. V. Authentication of herbal supplements using Next-Generation Sequencing. *PLoS One* **11,** e0156426 (2016).

208. Zerega, N. J. C., Mori, S., Lindqvist, C., Zheng, Q. & Motley, T. J. Using amplified fragment length polymorphisms (AFLP) to identify black cohosh (Actaea racemosa). *Econ. Bot.* **56,** 154–164 (2002).

209. Lum, M. R. *et al.* Identification of Botanicals and potential contaminants through RFLP and

sequencing. *Planta Med.* **71,** 841–846 (2005).

210. Lum, M. R. & Hirsch, A. M. in *Progress in Authentication of Food and Wine* 351–362 (ACS Symposium Series, 2011). doi:10.1021/bk-2011-1081.ch024

211. LeRoy, A., Potter, E., Woo, H.-H., Heber, D. & Hirsch, A. M. Characterization and identification of alfalfa and red clover dietary supplements using a PCR-based method. *J. Agric. Food Chem.* **50,** 5063–9 (2002).

212. Molina, J., Sherpa, C., Ng, J., Sonam, T. & Stuhr, N. DNA barcoding of online herbal supplements: crowd-sourcing pharmacovigilance in high school. *Open Life Sci.* **13,** 48–55 (2018).

213. Little, D. P. & Jeanson, M. L. DNA barcode authentication of saw palmetto herbal dietary supplements. *Sci. Rep.* **3,** 3518 (2013).

214. Baker, D. A., Stevenson, D. W. & Little, D. P. DNA barcode identification of black cohosh herbal dietary supplements. *J. AOAC Int.* **95,** 1023–34 (2012).

215. Little, D. P. Authentication of *Ginkgo biloba* herbal dietary supplements using DNA barcoding. *Genome* **57,** 513–516 (2014).

216. Michel, C.-I., Meyer, R. S., Taveras, Y. & Molina, J. The nuclear internal transcribed spacer (ITS2) as a practical plant DNA barcode for herbal medicines. *J. Appl. Res. Med. Aromat. Plants* **3,** 94–100 (2016).

217. Simmler, C. *et al.* Metabolite profiling and classification of DNA-authenticated licorice botanicals. *J. Nat. Prod.* **78,** 2007–22 (2015).

218. Marcus, D. M. Dietary supplements: What's in a name? What's in the bottle? *Drug Test. Anal.* **8,** 410–412 (2016).

219. Stoeckle, M. Y. *et al.* Commercial teas highlight plant DNA barcode identification successes and obstacles. *Sci. Rep.* **1,** 42 (2011).

220. Žiarovská, J., Rajchl, A., Fernández, E., Prchalová, J. & Milella, L. Identification of

Smallanthus sonchifolius in herbal tea mixtures by PCR and DART/TOF-MS methods. *Czech J. Food Sci.* **34,** 495–502 (2016).

221. Inglis, P. *et al.* DNA Barcoding for the identification of Phyllanthus taxa used medicinally in Brazil. *Planta Med.* **84,** 1300–1310 (2018).

222. Batista, J. M. M. *et al.* Molecular identification and phylogenetic analysis of Egletes viscosa (L.) Less., a traditional medicinal plant from Northeastern Brazil. *J. Braz. Chem. Soc.* **23,** 818–824 (2012).

223. Nakamura, S. S., Valle, J. S. do, Jacomassi, E., Linde, G. A. & Colauto, N. B. Molecular authentication of Maytenus sp by PCR-RFLP. *Semin. Ciências Agrárias* **34,** 627–634 (2013).

224. Palhares, R. M. *et al.* Medicinal plants recommended by the World Health Organization: DNA barcode identification associated with chemical analyses guarantees their quality. *PLoS One* **10,** e0127866 (2015).

225. Hoban, C. L. *et al.* Adulterants and contaminants in psychotropic herbal medicines detected with mass spectrometry and Next-Generation DNA sequencing. *Pharmaceut. Med.* **32,** 429–444 (2018).
